# Hashish Body Packing: A Case Report

**DOI:** 10.1155/2009/712573

**Published:** 2009-08-19

**Authors:** Manuel Jesus Soriano-Perez, Jose Luis Serrano-Carrillo, Inmaculada Marin-Montin, Alfonso Cruz-Caballero

**Affiliations:** ^1^Department of Internal Medicine, H.U.Virgen Macarena, 41007 Sevilla, Spain; ^2^Residencial Alta Entinas, Almerimar, El Ejido, C/Glaucio 4, 04711 Almeria, Spain

## Abstract

A 42-year-old African male was brought by the police to the emergency department under suspicion of drug smuggling by body-packing. Plain abdominal radiograph showed multiple foreign bodies within the gastrointestinal tract. Contrast-enhanced abdominal CT confirmed the findings, and the patient admitted to have swallowed “balls” of hashish. Body-packing is a recognized method of smuggling drugs across international borders. Body packers may present to the emergency department because of drug toxicity, intestinal obstruction, or more commonly, requested by law-enforcement officers for medical confirmation or exclusion of suspected body packing.

## 1. Case Report

**Figure 1:**
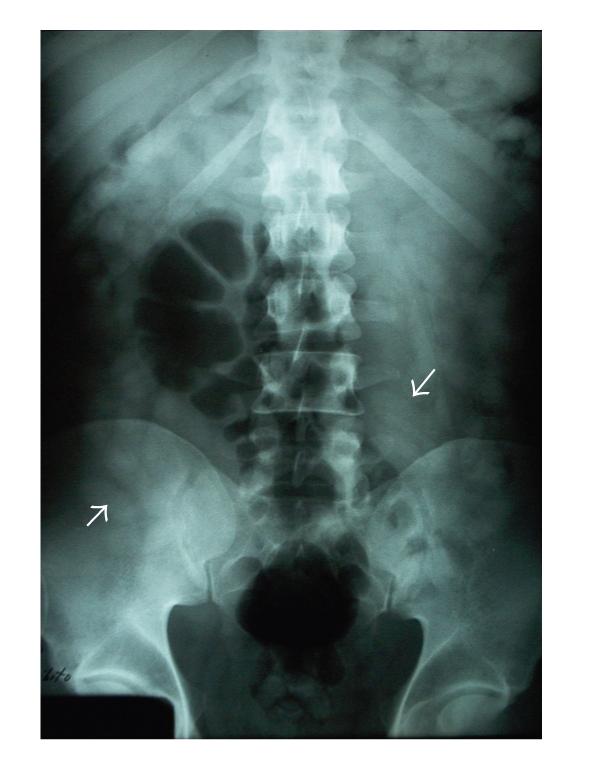
Multiple foreign bodies in the bowel in plain abdominal radiograph.

**Figure 2:**
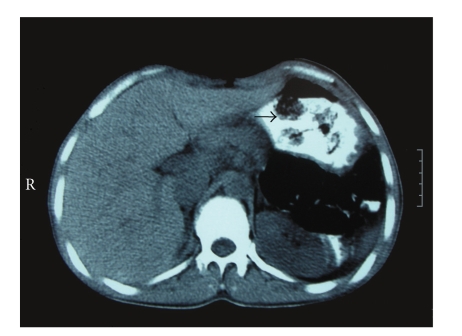
Oral contrast-enhanced CT showing rounded foreign bodies in stomach.

**Figure 3:**
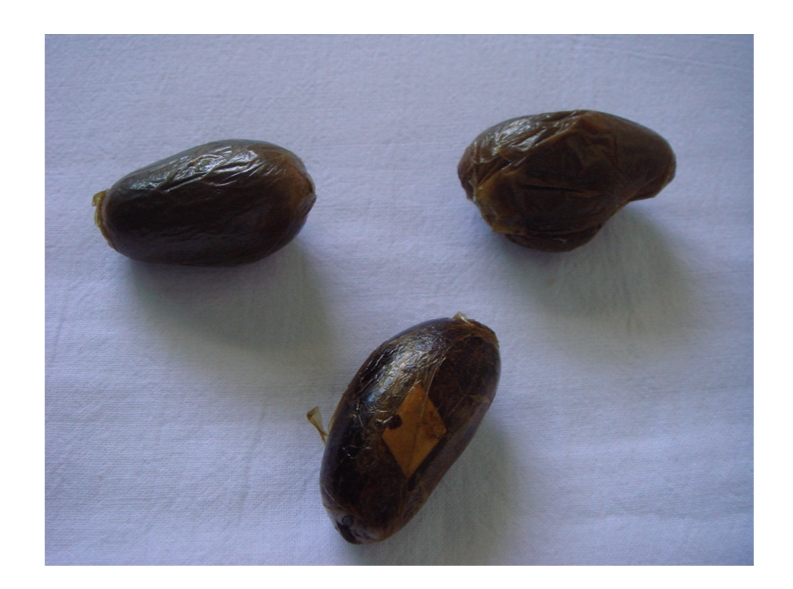
Hashish packages measuring approximately 5 cm each.

A 42-year-old African male was brought to the emergency department by the Spanish Police, after arriving at Seville airport, under suspicion of narcotics internal concealment (body-packing). On presentation, he appeared well with the exception of mild pain in the left iliac fossa. Physical examination was unremarkable. He was fully conscious and oriented. His blood pressure was 131/74 mmHg, the heart rate 83 beats per minute, respirations 14 per minute, and temperature 37.3°C. Cardiopulmonary, abdominal, and rectal examinations were normal, and there were no signs of drug intoxication or overdose. A plain abdominal radiograph showed multiple foreign bodies along the gastrointestinal tract (Figure 1). A contrast-enhanced abdominal CT confirmed the findings (Figure 2). The presence of more than fifty rounded foreign bodies from stomach to rectum was striking, and the patient admitted to have swallowed balls of hashish. After CT, the patient was given lactulose and sodium phosphate bowel preparation and was admitted to hospital for the next 7 days. During observation in hospital, the patient remained well and spontaneously passed 66 round packages, each measuring approximately 5 cm. (Figure 3). A second abdominal CT no longer demonstrated any intraluminal foreign bodies. He was discharged under the custody of law-enforcement authorities.

## 2. Discussion

Body-packing is a recognized means of international drug smuggling. The first case reported in the medical literature was in 1973, describing a patient who had swallowed a condom filled with hashish [1]. Body packing is mainly used for heroin, cocaine, amphetamines, and cannabis carriage. The packets can be made of various materials, but most often are latex condoms, which are easily available and can be swallowed or inserted into the rectum or the vagina in order to get across borders without being detected. 

Constipating agents, such as diphenoxylate or loperamide, are frequently used after swallowing the packets. After entering the country of destination, body packers use laxatives, cathartics, or enemas to help pass their cargo rectally [2]. 

 Occasionally, body-packers ingest more than one type of drug [3] and usually carry about 1 kg (2.2 lb) of drug, divided into 50 to 100 packets of 8 to 10 g each (0.3 to 0.4 oz), although persons carrying more than 200 packets have been described [4]. Each packet of cocaine, heroin, or amphetamine contains a potentially life-threatening dose of drug. 

Body packers may present to the emergency department because of drug toxicity, intestinal obstruction, or more commonly, requested by law-enforcement officers for medical confirmation or exclusion of suspected body packing [5]. 

A detailed history should be obtained. However, body packers are often unreliable historians, and in some cases, may be unable to provide a history owing to drug-induced toxic effects.

A plain abdominal radiograph is the initial method of choice for the detection or exclusion of drug-filled packets within the gastrointestinal tract of body packers. It may reveal multiple radiodense foreign bodies with unnatural uniformity, as in this case, a —rosette-like finding‖ formed by air trapped in the knot where a condom is tied, or a —double-condom‖ sign, in which air trapped between layers of latex making them more visible [6]. The last finding may also suggest a loss of integrity of the packing material. The radiopacity varies: hashish is denser than stool, cocaine appears similar to stool, and heroin has a gaseous transparency. Plain abdominal radiography has a sensitivity of 74%–100% [7]. The speed and safety of ultrasonography makes it appealing for the initial evaluation of body packers, but there are scant data in support of its use. Contrast-enhanced CT easily identifies drug packets, which typically appear as foreign bodies surrounded by a small amount of gas. CT is more sensitive than plain radiography, but sufficient assessment of sensitivity is lacking [8].

Management decisions depend on physical findings, type of drug, location of packets within the gastrointestinal tract, and type and size of packets. Uncommonly, drugs other than heroin or cocaine may have toxic effects after the packets leak or rupture [9].

The exact effect of hashish or marijuana on each person is unpredictable, since it depends on numerous factors including the individual's baseline personality, current psychological state, external conditions, previous experience of cannabis, the mode of use, and the quantity of Tetrahydrocannabinol (THC) taken into the body (no specific toxic or lethal dose has been clearly established thus far). Acute toxicity may result in tachycardia, postural hypotension, conjunctival injection, and ataxia. The pupils are unchanged, and sensorium is often clear. Psychiatric reactions include euphoria, anxiety, time-space distortions, fear, distrust, dysphoria, or panic disorder. Visual hallucinations and acute paranoid psychosis may occur with high doses. To our knowledge, there has never been a documented human fatality from cannabis overdosing via body-packing. Treatment of cannabis toxicity is supportive as there is no specific antidote.

Early surgery intervention was once recommended for asymptomatic body packers, probably because of the high rate of rupture of packets with primitive wrapping. The current approach to care, however, is to allow spontaneous passage of the packets with conservative therapy, including whole bowel irrigation, close observation, and careful monitoring in the hospital. The evacuation period, however, may last from 3 to 6 days. During this period, heroin packet leakage may require antidote therapy with the opioid antagonist naloxone. The overall rate of failure, defined as any indication for surgery, of such conservative management, is only about 5 percent [9]. This rate may actually be decreasing as packet production improves. Surgery may be indicated for patients with acute cocaine poisoning or gastrointestinal obstruction or perforation. Although successful endoscopic removal of packets from the stomach has been reported [10], the risk of packet rupture during the procedure has led others to caution against it.

## 3. Conclusion

We report a case of body-packing with hashish. The described patient did well with supportive care, clinical observation, and intestinal decontamination without adverse morbidity.
